# Case Report: A homozygous mutation in the *SPAG17* gene in a case with oligoasthenoteratozoospermic infertility

**DOI:** 10.3389/frph.2025.1554027

**Published:** 2025-04-22

**Authors:** Shruti Sethi, Waseem Andrabi, Kalyan Mitra, Singh Rajender

**Affiliations:** ^1^ Division of Endocrinology, Central Drug Research Institute, Lucknow, India; ^2^ Academy of Scientific and Industrial Research (AcSIR), Ghaziabad, India; ^3^ Nova Southend IVF Centre, New Delhi, India

**Keywords:** sperm morphology, SPAG17, genetic mutation, male infertility, oligoasthenoteratozoospermia (OAT)

## Abstract

**Background:**

Defects in sperm size and form, known as teratozoospermia, can adversely impair sperm motility and its ability to fertilize an oocyte. Teratozoospermia has been most often linked with genetic abnormalities with close to 100 genes known.

**Objective:**

The primary objective of this study was to investigate the genetic basis of oligoasthenoteratozoospermic infertility in an infertile man.

**Methods:**

We performed the whole exome sequencing, followed by *in silico* filtration of observed genetic variations. Filtered rare variants were assessed for their pathogenic nature on the basis of scores assigned by various in-silico tools and their biological relevance to sperm structural development. The potential pathogenic mutation was validated by Sanger sequencing.

**Results:**

Our study identified a homozygous substitution, c.4511A > G, in the *SPAG17* gene as a potential pathogenic mutation associated with oligoasthenoteratozoospermic infertility in the case under investigation. The mutation resulted in the substitution of asparagine with serine at the 1504^th^ amino acid position in a protein of 2,223 amino acids. This mutation shows a minor allele frequency of 0.0004671 in the gnomAD database. ACMG classification suggested this mutation to be likely pathogenic.

**Conclusion:**

Our study identified a homozygous likely pathogenic mutation (c.4511A > G, Asn1504Ser) in the *SPAG17* gene that explains oligoasthenoteratozoospermic infertility in the present case.

## Introduction

Infertility is described by the World Health Organization (WHO) as the inability to achieve pregnancy after at least 12 months of unprotected sexual intercourse. This condition has emerged as a significant reproductive health issue, affecting approximately 8%–12% of couples in the reproductive age group (1). More than 186 million individuals globally experience infertility, with the majority residing in developing countries (2). Male factor infertility stems from a wide range of causes, including genetic mutations, testicular dysfunction, systemic illnesses, medication use, or lifestyle choices. Genetic factors are the underlying cause in around 10%–15% of all male infertility cases (3). Male infertility is majorly represented by reduced sperm count (oligozoospermia), reduced motility (asthenozoospermia), and deformed sperm (teratozoospermia) or combinations of these. Teratozoospermia refers to an abnormality in sperm morphology that compromises the ability of spermatozoa to navigate through the female reproductive tract and fertilize an oocyte (4). These morphological defects include irregular head shapes, abnormal tail structures, and other deformities in spermatozoa (5).

The genetics of male infertility is highly complicated, involving the contribution of over 2,000 genes participating in the process of spermatogenesis (6). Mutations in close to 100 genes have been identified in cases with spermatozoal morphological defects (7). Despite this, the etiology underlying all sperm deformities remains yet to be understood. The investigation of the genetics of new cases of sperm deformities will add to the existing knowledge of the genetics of spermatozoal defects and the subsequent development of specific diagnostic tools and offering genetic counselling (8). In recent years, the advancement of next-generation sequencing (NGS) has greatly accelerated the identification of causative mutations in a cost-effective manner (9).

In this study, we present the genetic investigation in a patient with severe oligoasthenoteratozoospermia, leading to the identification of a potentially pathogenic homozygous mutation (c.4511A > G, Asn1504Ser) in the *SPAG17* (sperm-associated antigen 17) gene.

## Methodology

### Case details

A 35-year-old male diagnosed with oligoasthenoteratozoospermia was enrolled in this study. Detailed clinical investigation on his partner revealed no apparent sign of infertility. The couple achieved a successful pregnancy through intracytoplasmic sperm injection (ICSI) in the second attempt. The first attempt resulted in the formation of poor-quality embryos, leading to a failed outcome. A written informed consent was obtained from the patient before sample collection. The study was approved by the Institutional Human Ethics Committee of the Central Drug Research Institute (CDRI/IEC/2015/A1). The sample was obtained after following a 3-day abstinence period. The sperm count was performed manually using Makler counting chamber. For morphological assessment, slides were prepared using Hematoxylin and Eosin (H&E) stain (10). Sperm smears were prepared using 20 microliters of semen sample. The air-dried slides were sequentially dipped in 100%, 90%, 70%, and 50% ethanol for 15 min each. After treatment with 50% ethanol, the slides were placed under slow-running water for 15 min. The slides were then stained with hematoxylin for 1 min, followed by rinsing under running water for 15 min to remove excess stain. Eosin staining was performed for 1 min, after which the slides were again dipped in 70%, 90%, and 100% ethanol for 15 min each. The slides were subsequently cleared in xylene twice for 10 min. Finally, the slides were mounted in DPX and observed under a light microscope. The severity of sperm morphological abnormalities was determined following the WHO (2021) criteria, assessing 200 spermatozoa for head, midpiece and tail morphology, as well as the presence or absence of cytoplasmic droplets (11). Additionally, Scanning Electron Microscopy (SEM) was used to assess more detailed visualization of sperm surface morphology. Sperm motility was assessed manually, with total motility defined as the combined percentage of progressive and non-progressive spermatozoa. Teratozoospermia Index (TZI) was calculated by assessing the proportion of spermatozoa exhibiting multiple morphological anomalies (head, midpiece, or tail defects). The percentage of anomalies was recorded for each type of defect, and the TZI was calculated accordingly (12).

### DNA isolation and library preparation

Total DNA was extracted from sperm using the DNA MasterPure™ Complete DNA and RNA Purification Kit from Epicentre/Lucigen (Catalog number MC85200). The DNA integrity and yield were assessed by agarose gel electrophoresis and Qubit-based absorbance (Life Technologies Corporation, Q32850). Enzymatic digestion was carried out to obtain approximately 300 bp DNA fragments. Library preparation was undertaken using the MGIEasy Exome Universal Library Prep Kit (MGI, 1000009658).

### Exome sequencing

**Figure 1:**
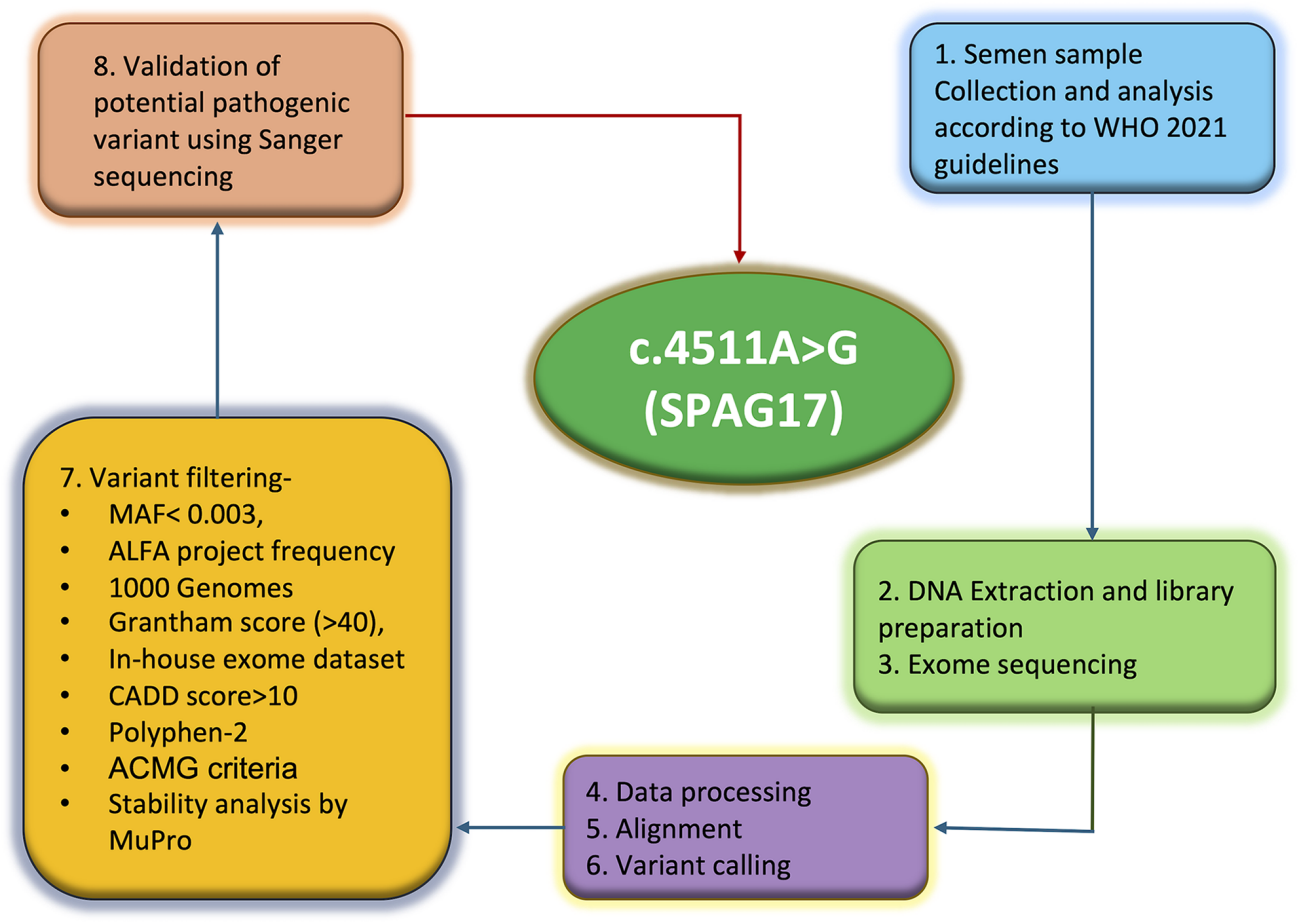
Graphical flowchart of the experimental plan.

Targeted exome-enriched DNA libraries were prepared using the SureSelect exome capture kit (Agilent Technologies), and the libraries were sequenced using the Illumina HiSeq 2500 sequencing platform. Base calling was done using GATK, and FASTQ format reads were aligned against the human reference genome build 19 using the Burrows-Wheeler Aligner (BWA) tool. The average coverage depth achieved for the sample was over 100X. SNVs and indel calling were done using GATK, and high-quality variants were annotated using Ion Reporter version 5.16 (Thermo Fisher Scientific, IR-42804). The complete methodology is illustrated in Figure 1.

### Variant selection and Sanger sequencing

The common variants were removed by applying a filter of 0.003 minor allele frequency (MAF). Additional filtration criteria also included Grantham score (>40) (13), PolyPhen-2 (>0.70) (14), PhyloP (≥0), and Combined Annotation Dependent Depletion (CADD) score (≥10). Furthermore, variants found to be common with 26 in-house exomes and three confirmed fertile control males were also excluded. This was followed by prioritizing the genes and variants on the basis of their established roles in spermatogenesis. Sanger sequencing employing primers specific to the mutation site in the *SPAG17* gene, forward (5'-3'): AGAACAGCAGGCCACACT and reverse (5'-3'): TTCTAACACCCACAGTGCCTAGC, was undertaken to validate the mutation.

### *In silico* mutation assessment

In-silico tools such as MUpro (15) and Dynamut2 (16) were used to assess the impact of the mutation on protein stability. Dynamut2 was used to generate a 3D model of the mutated protein to evaluate the effect of amino acid change on interaction with neighboring amino acids and the stability of SPAG17 protein. The substitution was also assessed as per the ACMG (American College of Medical Genetics) criteria (17).

## Results

### Semen parameter details

Semen analysis of the case under investigation showed a sperm concentration of 11 million per ml, total motility of 28% with progressive motility of 15%, and normal sperm morphology of 0.5%. Total motility refers to all sperm that were progressively or non-progressively motile. Progressive motility was not classified into type A or B. Sperm morphology assessment showed 99.5% of sperm having abnormal heads. Sperm head defects included amorphous head (46.5%), globular head (36%), pyriform head (15%), elongated head (1%) and double head (1%). Several tail defects were also observed, including coiled (61.5%), bent (3%), tailless sperm (3%), and hooked tails (2%). Out of the 200 spermatozoa assessed, 199 (99.5%) were identified as abnormal in at least one category. Of these abnormal sperm, 199 (100%) had head abnormalities, 139 (69.5%) exhibited tail abnormalities, and defects in the neck/midpiece or excess residual cytoplasm were also considered, but these abnormalities were not observed in this sample. SEM also shows minor artefacts around the neck or mid-piece regions, which could be due to procedural exposures adopted for SEM imaging (18). These defects were not included to avoid overemphasizing as these were inconsistent across sperm in the observation field, pointing to their likely origin during either sample preparation for SEM or image capturing. Often, sperm with head deformities were seen with bent or coiled tails (69.8%). The teratozoospermia index (TZI) for the patient was calculated to be approximately 1.69, indicating the presence of multiple defects in sperm morphology (12). This suggests that, on average, each abnormal sperm exhibited more than one structural defect, which highlights significantly poor sperm quality.

**Figure 2:**
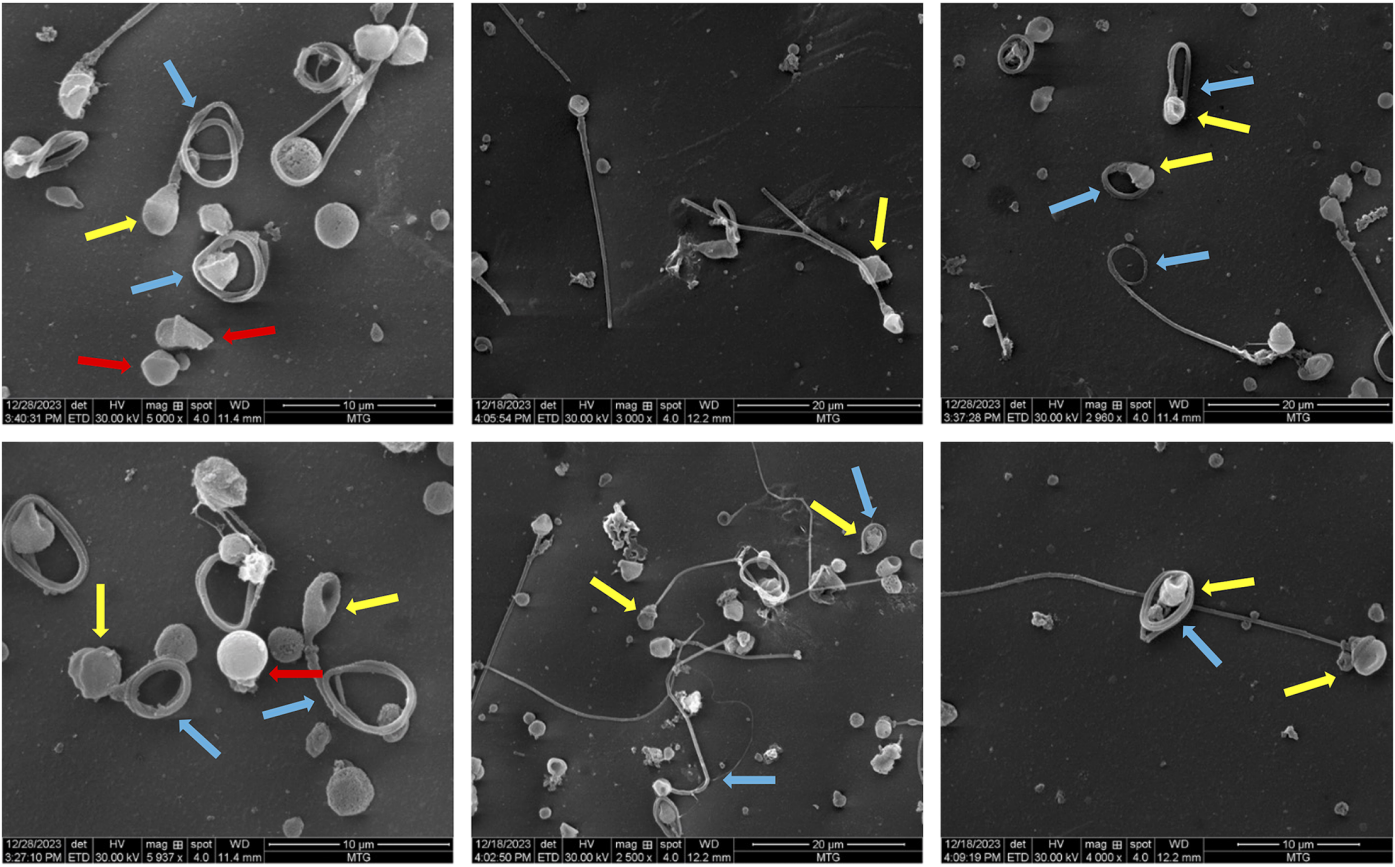
Scanning electron microscopy images showing various morphological deformities observed in the sample. The yellow arrows represent the abnormal collar-like structure around the sperm head; the blue arrows indicate tail abnormalities and the red arrows indicate acaudate sperm.

Scanning electron microscopy (SEM) analysis revealed an interesting morphological feature in spermatozoa. A prominent collar-like structure around the sperm heads was observed in 52% of the spermatozoa (Figure 2). The collar-like structure observed around the sperm head is included in the table under the “amorphous head” category as the term “collar-like structure” is not a standard term recognized by the WHO. This abnormality is primarily attributed to the defects in the development of round to elongating spermatids during spermiogenesis. It appears that the manchette, which usually disassembles and is removed during the formation of the sperm head, was only partially removed and remained there as a collar-like structure, causing defects in the proper sperm head morphogenesis. Morphological alterations observed in the case are listed in Table 1.

**Table 1:** Morphological abnormalities observed in the case.

Type of abnormality	Percentage
Tail	
Coiled tail	61.5
Bent tail	3
Hooked tail	2
Tailless sperm	3
Head	
Amorphous head	46.5
Globular heads	36
Pyriform heads	15
Elongated head	1
Double heads	1

### DNA library quality check

The quality and quantity assessment of DNA showed a concentration of 180 ng/μl with high purity and integrity. Library assessment and quantification were performed using a bioanalyzer from Agilent Technologies and TapeStation Analysis Software (version 4.1), which showed a library peak at 390 nucleotides. Sequencing on the Illumina HiSeq 2500 platform generated high-quality data (Table 2) with approximately 88,045 variants across the human genome.

**Table 2:** Quality scores of the whole exome sequencing data.

Parameter	Value
Total reads	89.32 Million
Total bases	8.93 Gb
Q20 bases	8.79 Gb (98.460820%)
Q30 bases	8.53 Gb (95.499967%)
GC content	42.14%

### Identification and validation of the mutation

**Figure 3:**
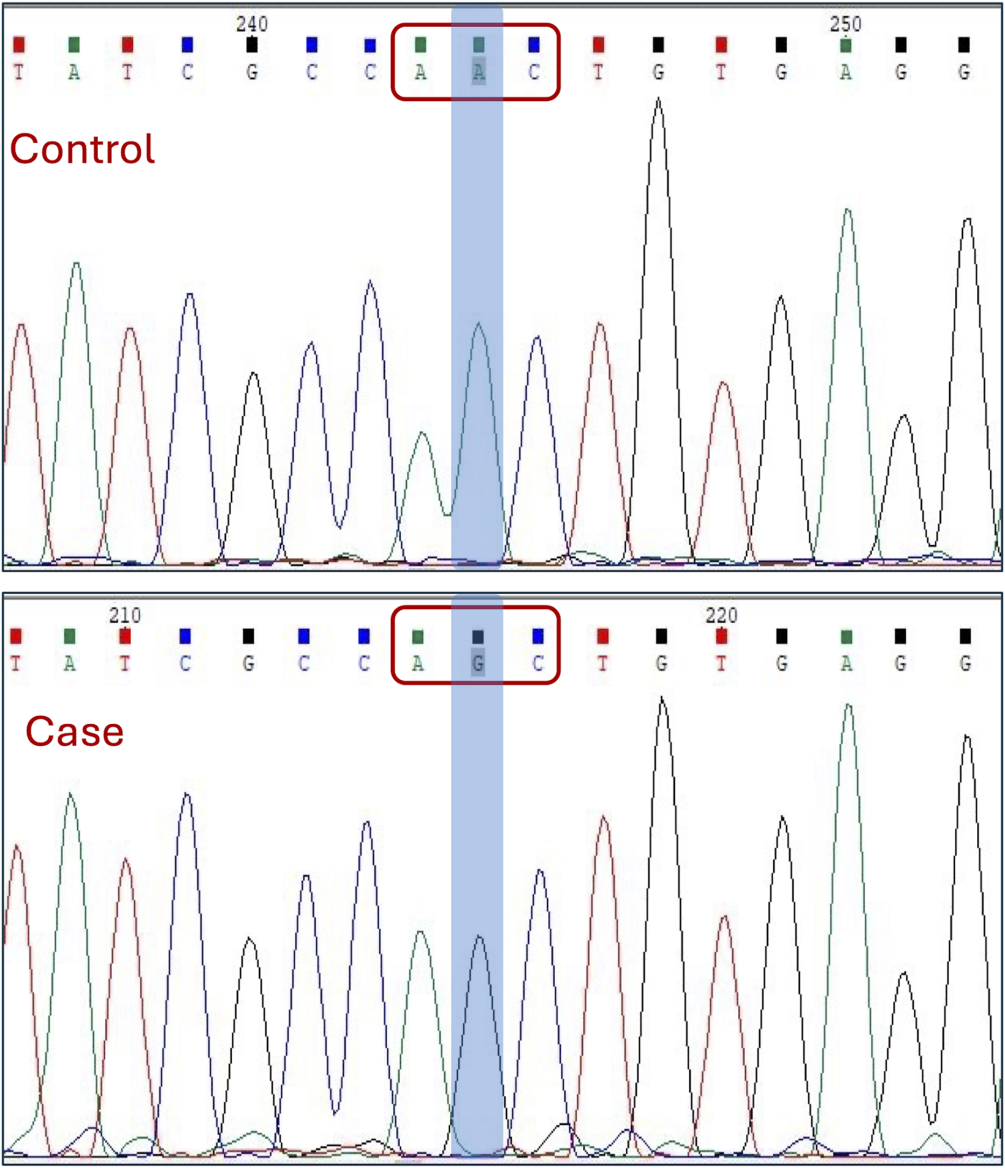
Sanger sequencing electropherogram showing the identified mutation in the SPAG17 gene. The codon and the exact nucleotide substitution are highlighted.

We first applied a filter of <0.003 MAF to eliminate common variants that are unlikely to be causative. Additional filters described in the methods section were used to narrow down to more meaningful mutation, followed by a search in the in-house exome data from unrelated samples and confirmed fertile controls. This sequential filtration narrowed us down to a pathogenic homozygous missense mutation, c.4511A > G (Asn1504Ser), in the *SPAG17* gene (Figure 3). Sanger sequencing was carried out to validate the mutation (Figure 3). Details of this mutation are provided in Table 3.

**Table 3:** Details of the pathogenic mutation in the *SPAG17* gene.

Locus	Genotype	Ref	Observed allele	Type	Variant ID	Coverage	Allele coverage	Allele ratio
chr1:118550743	C/C	*T*	*C*	SNV	rs542796610	26	*T* = 0, *C* = 26	*T* = 0.0, *C* = 1.0

**Figure 4:**
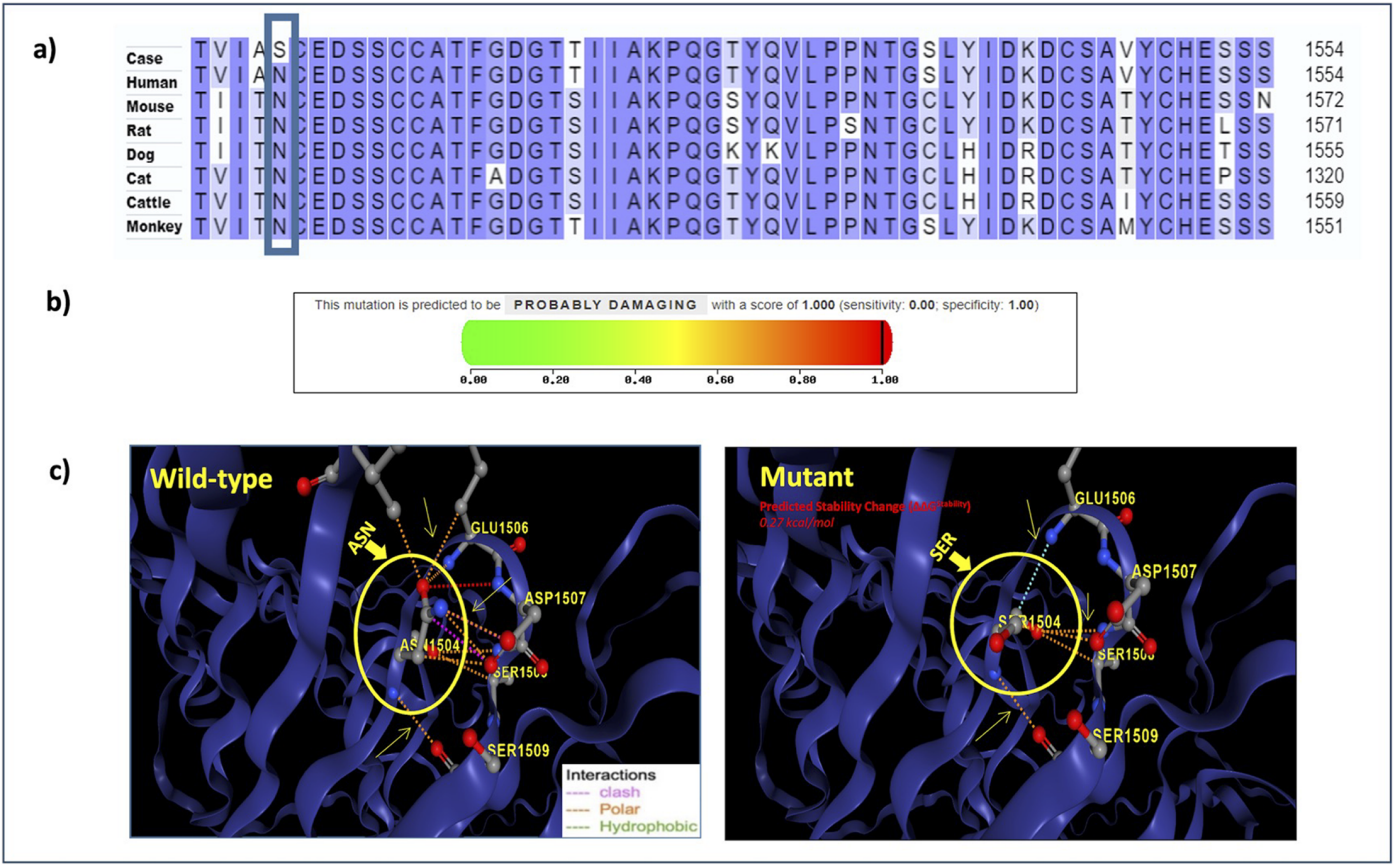
**(a)** Multiple sequence alignment reveals the conservation of the mutated residue across various mammalian species; **(b)** PolyPhen-2 report for *SPAG17* N1504S substitution: the black line denotes the position of the mutation on the severity scale ranging from 0 (green) to 1 (red), with higher scores indicating a more damaging effect; **(c)** structure of the SPAG17 protein; the circled region indicates the position of the reported mutation.

In human genome variation databases (19, 20), the allele frequency of the mutated allele (G) was reported to be very low in the general population (Table 4). This single nucleotide change resulted in the replacement of asparagine by serine (an aliphatic amino acid with a polar uncharged side group) at the 1504^th^ position in the SPAG17 protein. The conservation of asparagine amino acid across different mammalian species also suggested the potential pathogenicity of this non-synonymous substitution (Figure 4A).

**Table 4:** The frequency of the c.4511A > G mutation in various human genetic variation databases.

Population	Group	Sample size	Reference allele (*T*)	Altered allele (*C*)
ALFA project	Global	24,068	*T* = 0.99992	0.00008
ALFA project	Asian	202,312,233	1.00	0.0
1,000 Genomes	Global	5,008	0.9988	0.0012
1,000 Genomes	South Asian	978	0.994	0.006

### Variant pathogenicity assessment

The predicted pathogenicity scores for the c.4511A > G variant using different bioinformatics tools suggest its potential pathogenic nature (Table 5). The Grantham score of 46 suggests that amino acid properties would change substantially due to this mutation and suggests a potentially deleterious effect on the protein function. The PhyloP score of 5.56 implies that the mutation occurs in a more conserved nucleotide, making it potentially pathogenic. MUpro predicted a decrease in protein stability (*ΔG* = −1.4693494) that may result in a conformational change in the SPAG17 protein. A high PolyPhen-2 score supports a high probability of the variant being damaging (Figure 4B). The CADD score of 25 further supports the potential pathogenicity of the c.4511A > G variant.

**Table 5:** Pathogenicity scores assigned to the *SPAG17* c.4511A > G mutation by various *in silico* annotation tools.

Prediction tool	Score
Grantham	46
Polyphen-2	1
PhyloP	5.56
CADD	25
ACMG classification	PM2, PM6, PP2, PP3, PP4 (likely pathogenic)

The 3D structure of the SPAG17 protein developed using the DynaMut2 tool also showed the replacement of asparagine with serine to alter protein stability (Figure 4C) (16). The substitution resulted in alterations in a number of molecular interactions in the protein, including hydrophobic and polar interactions and the introduction of a number of steric hindrance. For example, the substitution of asparagine with serine at position 1504 altered the interaction between amino acids. The polar interaction (orange) and hydrogen bonding (red) between asparagine at 1504 and glutamic acid at position 1506 present in the normal protein were lost in the mutant, leading to the formation of new interactions, such as carbonyl bonds (blue) between serine at 1504 and glutamic acid at position 1506. Additionally, the clash-type (pink) interaction that was present between asparagine at 1504 and serine at 1508 in control was absent between serine at 1504 and serine at 1508 in the mutant, replacing it with the formation of a polar bond (orange). It suggests an altered stability in the mutant protein in comparison to the normal protein.

To sum up, each of the *in silico* prediction tools suggested a high likelihood of the c.4511A > G mutation to be pathogenic. The ACMG classification also suggested this mutation to be potentially pathogenic (Table 5).

## Discussion

Spermatozoa have characteristic structural features critical for motility and successful fertilization. The case under investigation showed a range of morphological abnormalities in the spermatozoa, including amorphous, globular, pyriform, elongated, and double heads, a distinctive collar-like structure around the sperm heads, and tail defects that included coiled, bent, and hooked tails. Whole exome sequencing identified a potential pathogenic mutation, c.4511A > G (Asn1504Ser), in the *SPAG17* gene. The *SPAG17* gene encodes a ∼250 kDa protein with 2,223 amino acids, playing essential roles in development and reproduction. Specifically, the protein encoded by the *SPAG17* gene is a key component of the central pair apparatus within the axoneme of the sperm flagellum. Multiple sequence alignment analysis revealed the evolutionary conservation of Asn1504 in the SPAG17 protein, which further supports the potential pathogenic nature of substitution at this site. Various *in silico* pathogenicity prediction tools suggested the selected variant to be damaging, and the ACMG criteria classified it as “likely pathogenic”.

*SPAG17* affects growth traits, such as skeletal development and body size in humans (21, 22). *SPAG17* mutations have been reported in primary ciliary dyskinesia in humans and mice (23, 24). A homozygous missense mutation in the *SPAG17* gene (exon 8: c.1069G > C; p.Asp357His) along with a mutation in the *WDR35* gene (exon 13: c.1415G > A; p.Arg472Gln) was linked to various brain and skeletal anomalies, including cranioectodermal dysplasia (CED) ciliopathy (25). Another homozygous loss-of-function mutation in the *SPAG17* gene, c.G4343A, which destabilizes the SPAG17 protein and reduces sperm motility was reported in a familial case of twins with severe asthenozoospermia (26). This showed that the production of SPAG17-deficient sperm is characterized by the development of flagellar defects and reduced motility, which is consistent with our findings. A recent study reported c.829 + 1G > T and c.2120del mutations in the *SPAG17* gene in four infertile men from two consanguineous Pakistani families, further strengthening the association of *SPAG17* mutations with sperm morphological abnormalities (27). These patients showed typical signs of Multiple Morphological Abnormalities of the sperm Flagella (MMAF), and a few axonemes were found to have a 9 + 1 arrangement with incomplete C1a projection. Spermatozoa exhibited a lower *SPAG17* mRNA level, with no protein detected along the sperm flagellum. Despite showing significant structural abnormalities in the sperm, patients didn't show any associated symptoms, such as respiratory or skeletal problems (27). We also did not observe any abnormality beyond spermatozoa.

*Spag17* knockout mice studies have reported both sperm head and tail defects. In the first study on *Spag17* knockout, skeletal defects, reduced mucociliary clearance, respiratory distress, and cerebral ventricular enlargement were observed (28). Another *Spag17* knockout study characterized sperm defects in more detail with specific head and tail defects (27). Specifically, the knockout mice exhibited infertility due to severe spermatogenic defects, characterized by arrest at the spermatid stage and the presence of abnormal sperm (many short flagella, irregular head shapes and almost no hook shape, unlike normal sperm) in the cauda epididymis. In addition, spermatozoa in the same knockout mice study showed abnormal manchette structure and aberrant microtubules, which were confirmed along with reduced chromatin condensation, irregular nuclear shape, and detached acrosome. The electron microscopy revealed that some axonemes lacked one of the central pair (CP) of the microtubule (9 + 1), whereas most comprised a 9 + 2 arrangement (29). This observation in mice relates to similar observations in human patients reported previously (27). Our findings are consistent with mouse knockout studies, which show that *SPAG17* knockout results in sperm morphological defects. In our case, SEM analysis uncovered a collar-like formation around the sperm head, suggesting abnormal differentiation during spermiogenesis. The manchette usually disassembles and is removed during sperm head maturation. The incomplete removal of the manchette during sperm development left a collar-shaped domain around the sperm head that resulted in improper head shape. Abnormal head forms that result from improper disassembly of manchette have been reported in infertility (30).

Interestingly, in addition to the gene knockout, specific mutant mice for the SPAG17 gene have also been produced. A novel hypomorphic allele of *Spag17* (c.5236A > T) was found to be responsible for the PCD phenotype in mice (23). This nonsense mutation introduced a premature stop codon (K1746*), which reduced the SPAG17 isoforms in mutant testes but not in the brain, showing that round spermatids did not fully mature and were unable to produce flagella (23). This implies that the *SPAG17* gene is required for spermiogenesis (23). This also suggests differential penetrance of specific mutations in comparison to complete gene knockout. Cryo-electron tomography of *Chlamydomonas* axonemes revealed that the two central microtubules differ in their protein composition, and PF6, an orthologue of the mammalian *SPAG17* gene in *Chlamydomonas*, is essential for the C1a projection of the axoneme, which plays a crucial role in flagellar motility (31). This explains the absence of one of the central microtubules in Spag17 knockout mice (29) and human patients (27). The above-mentioned investigations on *SPAG17* provide a strong linkage of this gene with sperm head and tail defects, reduced motility and specifically with the absence of one of the central pairs of microtubules.

We conclude that the observed sperm morphological defects, including collar-like structure on the sperm head and various tail abnormalities (coiled, bent and hooked), are due to the c.4511A > G mutation in the *SPAG17* gene. Our study adds to the growing body of evidence suggesting critical roles for the *SPAG17* gene in spermatogenesis and male fertility. SPAG17 knockout mice resulted in ramifications beyond sperm structural defects, which were seldom seen in human patients with specific mutations in the *SPAG17* gene. These reports suggest the involvement of SPAG17 in motile cilia development and its linkage with ciliary motility defects in sperm and other organs having motile cilia. The lack of appropriate functional assays to prove the effect of this mutation on sperm morphology is a limitation of the present study. Investigations like the current case study not only offer explanations for unique cases of infertility but also improve our understanding of the underlying molecular mechanisms of infertility that can potentially open new treatment avenues for male infertility.

## Data Availability

The original contributions presented in the study are included in the article/supplementary material, further inquiries can be directed to the corresponding author/s.
